# Cellular Pathophysiology of Leptospirosis: Role of Na/K-ATPase

**DOI:** 10.3390/microorganisms11071695

**Published:** 2023-06-29

**Authors:** Cassiano Felippe Gonçalves-de-Albuquerque, Carolina Medina Coeli da Cunha, Léo Victor Grimaldi de Castro, Caroline de Azevedo Martins, Marcos Roberto Colombo Barnese, Patrícia Burth, Mauricio Younes-Ibrahim

**Affiliations:** 1 Laboratory of Immunopharmacology, Department of Physiology, Federal University of the State of Rio de Janeiro (UNIRIO), Rio de Janeiro 20211-030, Brazil; cmedina@id.uff.br; 2 Neuroscience Graduate Program, Federal Fluminense University (UFF), Niteroi 24000-000, Brazil; 3 Cellular and Molecular Biology, Oswaldo Cruz Institute (IOC), Rio de Janeiro 21040-900, Brazil; leovictorgricastro@hotmail.com; 4 School of Medicine and Surgery, Federal University of the State of Rio de Janeiro (UNIRIO), Rio de Janeiro 20270-901, Brazil; caroline.martins@unirio.br; 5 FISCLINEX Postgraduate Program, State University of Rio de Janeiro (UERJ), Rio de Janeiro 20550-900, Brazil; marcos.robertosh@gmail.com; 6 Laboratory of Enzymology and Cellular Signaling, Department of Cellular and Molecular Biology, Federal Fluminense University (UFF), Niteroi 24000-000, Brazil; pburth@id.uff.br; 7 Department of Medicine, Pontifical Catholic University of Rio de Janeiro (PUC-Rio), Rio de Janeiro 22453-900, Brazil; 8 State University of Rio de Janeiro (UERJ), Rio de Janeiro 20550-900, Brazil

**Keywords:** leptospirosis, Na/K-ATPase, glycolipoprotein, GLP

## Abstract

Inada and Ido identified *Leptospira* sp. as the pathogen responsible for Weil’s Disease in 1915. Later, it was confirmed that Leptospira causes leptospirosis. The host microorganism’s interaction at the cellular level remained misunderstood for many years. Although different bacterial components have been isolated and purified, the complexity of the molecular interactions between these components and the host and the molecular mechanisms responsible for the systemic dysfunctions still needs to be fully unveiled. Leptospirosis affects virtually all animal species. Its cellular pathophysiology must involve a ubiquitous cellular mechanism in all eukaryotes. Na/K-ATPase is the molecular target of the leptospiral endotoxin (glycolipoprotein—GLP). Na/K-ATPase dysfunctions on different types of cells give rise to the organ disorders manifested in leptospirosis. Concomitantly, the development of a peculiar metabolic disorder characterized by dyslipidemia, with increased levels of circulating free fatty acids and an imbalance in the fatty acid/albumin molar ratio, triggers events of cellular lipotoxicity. Synergistically, multiple molecular stimuli are prompted during the infection, activating inflammasomes and Na/K-ATPase signalosome, leading to pro-inflammatory and metabolic alterations during leptospirosis. Leptospirosis involves diverse molecular mechanisms and alteration in patient inflammatory and metabolic status. Nonetheless, Na/K-ATPase is critical in the disease, and it is targeted by GLP, its components, and other molecules, such as fatty acids, that inhibit or trigger intracellular signaling through this enzyme. Herein, we overview the role of Na/K-ATPase during leptospirosis infection as a potential therapeutic target or an indicator of disease severity.

## 1. Introduction

Leptospirosis is a zoonosis with a worldwide distribution and greater prevalence in tropical and temperate regions and emerging countries, such as those in South and Southeast Asia, Oceania, the Caribbean, Latin America, and sub-Saharan Africa, which have the highest mortality and morbidity from the disease. Outbreaks hit Cuba, Brazil, India, Hawaii, and Puerto Rico hardest. It has been estimated that more than one million cases and almost 59,000 deaths occur annually worldwide [1,2]. Human leptospirosis is clinically pleomorphic, and mortality is higher in elderly patients who develop the icteric form of renal failure and is lower in younger anicteric patients. In addition, bacterial pathogenic factors may be associated with a higher mortality rate.

The transmission of leptospirosis occurs mainly through the urine of infected animals, mainly rats, which are the main reservoirs of the bacteria. Indirect human exposure occurs through contaminated soil, water, or food. Leptospira survives in water or moist soil for long periods and actively penetrates the host through mucosa or injured skin [3,4]. Low socioeconomic status is directly linked to greater vulnerability to infection by leptospirosis. People easily exposed to sewage water, accumulated garbage, and rat infestation have a greater chance of becoming infected [5]. Heavy rains and floods strongly contribute to outbreaks in endemic areas, linked to poor sanitation and infrastructure conditions [6,7]. Occupational risk factors such as those associated with agriculture and military work affect mainly male patients [8].

The clinical course of leptospirosis is often biphasic: leptospiremia initially, followed by the immune phase. Symptoms include fever, chills, headache, myalgia, nausea, vomiting, and diarrhea. The presence of jaundice and kidney failure configures icterohemorrhagic leptospirosis (Weil’s Disease). The hepato-renal-pulmonary manifestation is the most severe form. Ocular symptoms, hepatitis, pancreatitis, meningitis, and pulmonary changes may also occur [2]. For the diagnosis, the most sensitive and specific technique for the acute phase of the disease is polymerase chain reaction (PCR), but it is not an easily accessible method. The most used are serological methods such as microagglutination (MAT), rapid tests, and immunoenzymatic assay (ELISA), which can detect IgM as early as 5 to 7 days after the onset of symptoms, up to weeks or months after the cure of the disease. Dark-field microscopy and isolation of leptospira from blood, urine, or other clinical materials may be available in some reference centers [9]. Briefly, treatment consists of symptom control, hydration, correction of electrolyte disturbances, and antibiotic therapy with doxycycline or penicillin [10,11]. Herein, we intend to review the role of Na/K-ATPase in leptospirosis pathogenesis, including its molecular targets and endotoxins, key concepts, and some chronological timelines.

## 2. Leptospira—A Particular Type of Bacteria

In 1907, Stimson found spirochetes in the renal parenchyma of autopsy study subjects whose deaths were attributed to yellow fever. In 1915, Inada and Ido [12] inoculated blood from a patient with Weil’s disease into guinea pigs and experimentally reproduced the disease, revealing the bacterial etiology of leptospirosis. In 1918, Noguchi [13] identified the morphological characteristics of this bacterium and defined the new genus Leptospira (lepto = thin, spire = ball).

Leptospira is a motile, aerobic bacterium that divides by binary fission, is not Gram stained, and has a long and spiral form. Leptospira has Gram-negative bacteria characteristics, such as a double membrane and lipopolysaccharides (LPS). However, it differs from typical Gram-negative bacteria, with the peptidoglycan layer adhering to the inner membrane [14]. The bacterium measures between 0.1 and 1.5 µm in diameter and 3 to 20 µm in length. It has a periplasmic flagellum that inserts into each end of the protoplasmic cylinder used by the bacteria to move. The protoplasmic cylinder is formed by the cell wall, membrane, and cytoplasmic content [15]. The outer envelope, a coating composed of proteins, lipopolysaccharides, and phospholipids, covers the protoplasmic cylinder and axial filament [16,17]. Genome analysis of *Leptospira interrogans* (pathogenic) serovar Icterohaemorrhagiae showed two chromosomes, with 4768 predicted genes [18], and *Leptospira biflexa* (not pathogenic) sequencing also revealed two chromosomes, with 3590 protein-coding genes [19].

Leptospira bacteria are part of the bacteria phylum called spirochetes. About 64 species of Leptospira are classified as saprophytic or pathogenic [20]. Among the 66 species considered pathogenic [20,21,22,23], *L. interorgans* stands out, with Icterohaemorrhagiae and Copenhageni as the most relevant serovars [2]. Leptospira is aerobic or microaerophilic, and, instead of carbohydrates or amino acids, it uses long-chain fatty acids as an energy source. In addition, it is a bacterium that grows slowly in culture and needs a specific medium that contains serum or serum albumin and Tween 80 polysorbate EMJH (Ellinghausen–McCullough–Johnson–Harris) [24,25]. The ideal temperature for growth is around 30 °C, and the pH ranges from 6.8 to 7.4.

## 3. Outer Envelope Toxins

Leptospira’s outer envelope (OE) comprises proteins, lipids, and carbohydrates that correspond to about 15% of the dry weight of the bacterium. The OE lipid fraction of the Copenhageni serovar is composed of 80% phospholipids (of which 98% is phosphatidylethanolamine) and 20% free fatty acids (myristate 2.9%, palmitate 17.5%, palmitoleate 25%, stearate 1%, and oleate 51.5%) [26,27]. The OE is composed of LPS, OmpL1 (porin protein), and numerous lipoproteins such as Leptospira lipoprotein 32 (LipL32), LipL41, LipL21, Leptospira ompA domain protein 22 (Loa22), and repeat A, similar to Leptospira immunoglobulin (LigA). These proteins are essential virulence and pathogenicity factors of the bacterium and may play an essential role in its furtive character [28]. The OE highlighted the different antigenic fractions capable of inducing a protective immune response in the hamster, enabling the development of vaccines [29]. The fractions described below were extracted from the OE.

### 3.1. Lipopolysaccharides (LPS)

Lipopolysaccharides are present in the OE and represent 3 to 5% of the dry weight of the bacteria [30,31]. Lipopolysaccharides (LPS) from leptospira are different from those classically described in Enterobacteriaceae because of their structure and biological activity [32,33]. For this reason, they are called “lipopolysaccharide-like” lipopolysaccharides (LLS). Homogeneously distributed in the OE [26], LLS are considered the primary determinant antigens of the microagglutination test. Because of these antigenic properties, LLS are also inducers of natural immunity [34] and are used for active immunization [35,36,37]. As LLS isolated from different leptospiral serotypes have distinct electrophoretic and biological properties, the immunizations are serotype specific [27,36]. The leptospiral LPS present a composition of carbohydrates and lipids different from the LPS from typical Gram-negative bacteria, with hydroxylauric, palmitic, and oleic acids being the primary fatty acids found [26]. In addition, LLS from pathogenic Leptospira differ from those from non-pathogenic or intermediate pathogenic ones. The chemical composition of LLS from *L. interrogans* serovar Copenhageni has an extended O-antigenic polysaccharide consisting of sugars not present in serovar Varillal from *L. licerasiae* and also presents hydroxypalmitate, not found in other species [38]. Leptospira LLS do not activate macrophages via (toll-like receptor 4) TLR4; instead, they activate via TLR2 [39]. Lack of activation via TLR4 impairs TRIF- and RANTES-dependent nitric oxide production. In addition, critical antimicrobial responses and the O antigen appear to contribute to this failure [40]. Another consequence of impaired TLR4–TRIF signaling is reduced CD40 expression in dendritic cells (DC), a vital component for generating memory B cells [41]. The leptospiral LLS do not seem to have pyrogenic potential and have not been proved cytotoxic [31].

### 3.2. The Lipid A

Lipid A isolated from Leptospira is neither toxic nor pyrogenic, and its mitogenic power is lower than that seen in other bacteria [42,43]. Like LPS from Gram-negative bacteria, LLS from Leptospira can activate macrophages and stimulate the production of interleukin-1 and interferon [32].

### 3.3. Proteins

Different proteins with molecular weights ranging between 22 and 42 kD have been detected in the OE [44,45]. Unlike LLS, which are immunologically specific for their serovars, these proteins cross-react with proteins from serovars and even with those from other serogroups [44,46]. The importance of these proteins in the virulence and pathogenesis of leptospiras remains unknown.

### 3.4. LipL32 and HlyX

LipL32 is a surface antigen of pathogenic leptospiral species. HlyX is a protein expressed only in pathogenic strains. Leptospira, unlike other pathogenic bacteria, does not have its peptidoglycans (PG) recognized by NOD1 and NOD2 receptors. LipL21 seems to bind to PG more strongly in pathogenic Leptospira, thereby protecting PG from degradation in muropeptides and preventing recognition by NOD [47]. LipL32 is the most abundant surface protein in pathogenic leptospira and is highly immunogenic [48,49]. LigA and LigB also contribute to leptospiral adhesion to the extracellular membrane, binding to collagen I and IV, laminin, fibronectin, and fibrinogen [50]. OmpL1, on the other hand, plays a significant role in adhesion to mammalian cells through binding with glycosaminoglycans [51].

### 3.5. Glycolipoprotein (GLP)

A glycolipoprotein (GLP) fraction was extracted from the OE of *L. interrogans* (serogroup Icterohaemorrhagiae, serotype Copenhageni) [26]. It comprised proteins, lipids, and carbohydrates in almost equivalent proportions, representing 3 to 5% of the leptospira weight. With this GLP fraction, the authors demonstrated “in vitro” cytotoxic effects induced by a Leptospira extract for the first time. In the experiment, the incubation of mouse fibroblasts with GLP promoted the appearance of numerous cytoplasmic vacuoles, followed by the destruction of plasma and nuclear membranes. These effects were dose dependent and increased proportionally with incubation time. Pre-treatment of GLP with proteinases and lipases also made it possible to incriminate the lipid component of this GLP as being responsible for this cytotoxic effect. So, GLP appears to produce cytotoxic effects on host cells, contributing to the pathogenesis of acute leptospirosis.

This toxic action produced by GLP occurs in all leptospiral species, showing that pathogenicity depends on other factors besides toxicity [26]. Other studies showed the ability of a *L. interrogans*-derived GLP to activate monocytes and induce secretion of tumor necrosis factor-alpha (TNF-α), interleukin-10 (IL-10), and IL-6 and increase CD69 expression on T lymphocytes and monocytes, which may contribute to the release of other inflammatory mediators such as prostaglandins, B4 leukotrienes, and nitric oxide. In contrast, GLP from non-pathogenic *L. biflexa* did not have the same effect. Furthermore, it was observed that GLP did not block LPS from binding to these cells, suggesting that activation is induced by a different pathway [52,53,54], as well as having a synergistic action with LPS to induce the production of IL-1β via the NLRP3 (NLR family pyrin domain containing 3) inflammasome [55].

Working with the same bacterial extracts described by Vinh et al. (1986) [25], GLP also inhibited the activity of different isoforms of the Na/K-ATPase, present in the brain, kidney, liver, and lung in different animal species [55]. This dose-dependent effect validated the initial hypothesis of a ubiquitous molecular mechanism awakening different functional aspects resulting from the impact of this cellular phenomenon in the different systems of the organism. These new approaches place GLP as a highly relevant endotoxin for the pathogenesis of leptospirosis, mainly due to its effect on the different epithelial cells of the organism [56,57].

## 4. Pathogenesis

Leptospira penetrates the host and effortlessly reaches the bloodstream, promoting bacteremia. The bacteria reach organs and tissues through circulation, colonizing the spleen, liver, lungs, and kidneys, where they multiply and spread [58]. The highest load of leptospira in the blood can be detected on the fifth day after infection [59].

Leptospirosis usually has an incubation period of 5 to 14 days but can reach up to 30 days. From there, the infection can manifest itself in two ways: anicteric or icteric. Next, comes the immune phase, when the bacteria are no longer found in the blood. The severe icteric form, called Weil’s Syndrome, involves bleeding disorders, jaundice, and acute renal failure [60,61].

Bleeding disorders can be explained by the hemolytic and cytotoxic action of hemolysin SphH, a leptospiral protein capable of forming pores in several mammalian cells, with disruption of the cell barrier and development of hemorrhages [62]. In the liver, leptospirosis disorganizes hepatocytes with disruption of intercellular junctions, resulting in moderate elevation of liver transaminases and direct bilirubin cholestasis, causing jaundice.

Leptospira colonizes the renal tubules in the kidneys, promoting lymphocyte infiltration [63,64]. Kidney damage is clinically manifested with acute kidney injuries and electrolyte changes, such as hypokalemia, natriuresis, magnesium loss, and an abrupt decrease in creatinine clearance. The detection of GLP in tissues evidences the importance of this endotoxin in the virulence and pathogenesis of Leptospira [63,65,66,67].

Through clinical trials, the hypokalemia of leptospirosis was investigated to clarify whether the low potassium levels in the blood of patients with leptospirosis is due to the redistribution of potassium into the cells. Based on serum and intraerythrocytic potassium levels, low concentrations were found in both the extra- and intracellular compartments [68]. Furthermore, patients with acute renal failure (ARF) have lower levels of intraerythrocytic potassium than those without ARF, suggesting that this feature may be related to the severity of the disease [69]. Patients with leptospirosis and ARF studied by high-resolution electrocardiograms showed ventricular electrophysiological changes, with late ventricular potentials in the acute phase of the disease, reversible with recovery [70]. This in vivo event was compatible with the inhibiting Na/K-ATPase activity induced by GLP in cardiac tissue, as demonstrated by in vitro tests [71].

Involvement of the skeletal muscles is precocious and very marked in leptospirotic infection; myalgias are intense and generalized and can evolve with rhabdomyolysis, the necrosis of muscle cells (116,197). Histopathological examination of skeletal muscles of patients with ARF shows the presence of heterogeneous cellular lesions with an irregular pattern of cytotoxicity, whose morphological consequences vary in the same microscopic field from edema to myocyte necrosis.

## 5. Pathophysiology—Na/K-ATPase and GLP—A Molecular Target

Leptospirosis is a systemic disease that affects several organs and presents peculiar hydroelectrolytic disturbances. Therefore, the pathophysiology of the disease necessarily involves ubiquitous cellular mechanisms. Our group investigated the role of the Na/K-ATPase ion transport enzyme as a target for the leptospira endotoxin. Na/K-ATPase is a transmembrane protein that, through ATP hydrolysis, maintains the cell’s electrical potential and intracellular water volume, pumping two potassium ions into and three sodium ions out of cells [72]. This enzyme is a membrane protein in all eukaryotic cells and is essential for proper cell physiology. Molecules such as thyroid hormones, corticosteroids, epinephrine, norepinephrine, and insulin can interfere with the activity of Na/K-ATPase [73]. Some molecules block its activity, such as ouabain, which was initially considered a specific inhibitor, but can no longer be considered as such [17]. By Jorgensen’s method [74], with purified NA/K-ATPases from rabbit brains and kidneys, GLP inhibited Na/K-ATPase in a dose-dependent manner, confirming that all the different isoforms of the protein are sensitive to GLP. The α1 isoform of the kidney-derived enzyme is slightly less sensitive to GLP than the α2 and α3 isoforms derived from brain tissue [56,75].

**Figure 1:**
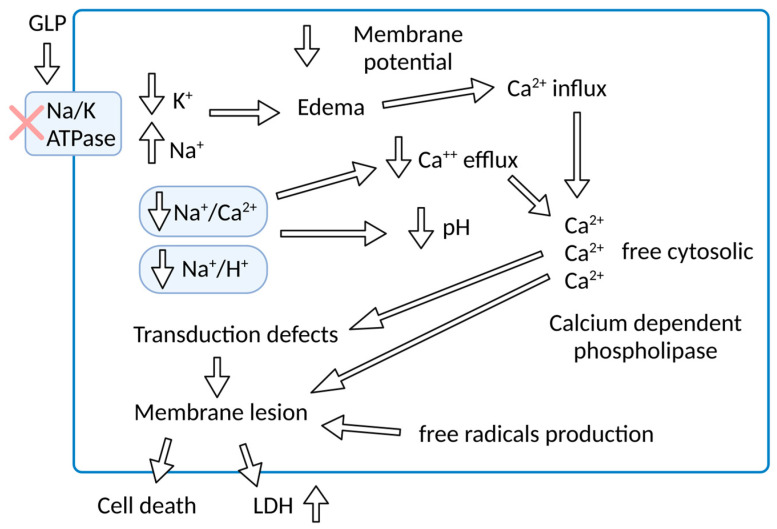
Schematic representation of the effect of GLP on Na/K-ATPase activity with pathophysiological consequences for renal epithelial cells.

To evaluate the interaction of GLP with Na/K-ATPase in different segments of the nephron of rabbits and rats, microdissections of the proximal convoluted tubule (PCT), medullar thick ascending limb (mTAL), and the cortical collecting duct (CCD) were performed. Both in segments of rats (species resistant to leptospirosis) and rabbbits, mTAL showed greater sensitivity to GLP than PCT and CCD, with no difference in sensitivity between the two species [56] (Figure 1). These observations indicate that the natural resistance of a given animal species to leptospirosis is not due to the lack of sensitivity of the Na/K-ATPase of its cells to GLP but to the bioavailability of this endotoxin in the tissues of the infected animal. This means that the level of colonization of the bacteria in the tissue is a crucial factor in determining the animal’s vulnerability. Other studies reinforced this consideration, such as when resistant mice induced to immunosuppression become susceptible to leptospirosis [76].

Most Na/K-ATPase inhibitors act as competitive inhibitors with potassium, with an effect similar to that promoted by ouabain. The effect of GLP on the affinity of sodium and potassium ions, on the contrary, occurs with an increase in the affinity of the enzyme for sodium [75]. The effect of GLP on intact renal tubular cells was also tested through the influx of rubidium Rb^+^ [77]. The result confirmed that GLP also reduces the activity of Na/K-ATPase in intact cells. 

With the verification of the effect of GLP on Na/K-ATPase in vitro, interest in its specificity intensified. The effect of GLP on other membrane proteins, such as Mg-ATPase and adenylcyclase in kidney cells from rats and rabbits, was tested. GLP did not alter the activity of these enzymes, confirming the specificity of GLP for Na/K-ATPase. We also evaluated whether the Na/K-ATPasic activity is modified by the presence of LPS extracted from *L. interrogans* serovar Copenhageni or *L. interrogans* serovar Hebdomadis, *Escherichia coli,* and *Salmonella minnesota*. None of these preparations affected the enzymatic activity [56,78].

To understand the active principle of the GLP fraction, it was verified through membrane filtration (10 kDa cut-off membrane) that the inhibitory fraction is retained, and its effect remains even after the boiling process and proteolytic treatment. In conclusion, the molecular weight of the active principle is greater than 10 kDa, and it is not a protein fraction [78]. Finally, the Na/K-ATPase inhibition ranges of the intact GLP and the lipid fraction extracted from GLP were very close, confirming that the active principle resides in the lipid fraction of GLP [78].

The following step was to define the nature of this lipid fraction. After separation of polar lipids and non-esterified fatty acids (NEFA), only the NEFA fraction (15 to 20% of the total lipids in GLP) remained, inhibiting Na/K-ATPase. With better purification and separation of NEFA, two peaks capable of inhibiting the enzyme were evidenced, corresponding to palmitoleic and palmitovacenic acids and octadecenoic acid (oleic acid) [78].

An attractive characteristic was the addition of albumin or ovalbumin in the experiments, annulling the inhibitory effect of Na/K-ATPase by GLP [56]. This is consistent with albumin’s adsorption effect on fatty acids (FA). Albumin transports free FA throughout the body [79]. These findings suggest that serum albumin may be a protective factor against the lipotoxic effect of GLP, especially in the leptospiremic phase of the disease. This may even be related to detoxification and leptospiral endotoxin overload in target organs, such as the liver and kidney [80].

The pulmonary alveoli’s water and electrolyte reabsorption systems depend on their cells’ structural and functional integrity in the lung epithelium. Active sodium transport is carried out from the lumen of the alveoli to the pulmonary interstitium. It generates an osmotic gradient that constitutes the driving force for the reabsorption of alveolar fluids [81,82,83,84]. The absorbed fluids are drained by the lymphatic blood vessels, ensuring the viability of the alveolar lumen. Type II alveolar cells, or type II pneumocytes, are responsible for most alveolar–interstitial sodium vector transport. These cells have a sodium channel in the apical membrane and the enzyme Na/K-ATPase in the basolateral membrane [83], so the role of Na/K-ATPase in type II pneumocytes as a conductor of alveolar–interstitial sodium and water flow is well established [12].

## 6. Organ Impact of Molecular Mechanisms

Na/K-ATPase is the molecular machinery responsible for pumping Na^+^ and K^+^ across cell membranes, but it is also an essential mediator of cell transduction and plays a crucial role in cell physiology [85]. In leptospirosis, the inhibition of Na/K-ATPase induced by GLP is a mechanism potentially responsible for the cellular pathophysiology in several organs, also manifested by disturbances in the electrolyte balance between organic compartments [56]. Acute kidney injury with paradoxical hypokalemia and body loss of sodium and magnesium frequently occurs in human leptospirosis [86]. Another target organ of leptospirosis is the liver, resulting in hemorrhagic manifestations, liver dysfunction, and cholestatic jaundice. Biliary secretion also depends on the sodium gradient generated by the Na/K-ATPase enzyme in the bile canaliculi [86]. In the lungs, leptospirosis can cause intra-alveolar hemorrhage and acute respiratory distress syndrome (ARDS) [87]. GLP inhibits alveolar Na/K-ATPase and can induce ARDS. Cardiac inhibition of Na/K-ATPase can cause cardiac arrhythmia, which can be detected in vivo in patients with leptospirosis by electrocardiogram. Intestinal effects of leptospirosis include diarrhea. Enzyme inhibition in skeletal muscle can cause rhabdomyolysis, and, in severe forms, systemic and inflammatory involvement can promote multi-organ failure [56].

Dyslipidemia is also seen in leptospirosis, such as increased plasmatic concentrations of oleic acid (OA), synergistically an inducing factor of lung injury, inhibiting alveolar sodium transport. However, Na/K-ATPase does not appear to be the only molecular target of OA, which occupies additional targets in cell membranes, such as receptors and fatty acid transporters [84].

Based on previous reports that increased OA plasma concentrations induce lung injury by interfering with sodium transport, OA being one of the active lipid components functioning as Na/K-ATPase inhibitors in GLP, a new non-radioactive assay to quantify Na/K-ATPase in vivo was proposed, utilizing OA injections. OA administration was used with and without ouabain as a comparison factor because of its well-described effect as an Na/K-ATPase inhibitor. Results reinforced that Na/K-ATPase inhibition is crucial in lung injury during high plasma OA levels, as in leptospirosis [88]. Due to the critical role of OA in several pathologies, its effect on Na/K-ATPase activity was compared with the lung Na/K-ATPase inhibition caused by ouabain. Previous reports showed that the ouabain and OA had similar in vivo effects on the Na/K-ATPase, probably because of higher OA doses. However, Na/K-ATPase is not the sole target for OA. Ouabain is more effective in lung Na/K-ATPase inhibition than OA, suggesting that OA should have additional targets in cell membranes, such as fatty acid receptors and fatty acid transporters [89].

In summary, after organ colonization, leptospira lysis occurs, caused by the host immune response, which releases GLP, a potent and specific Na/K-ATPase inhibitor. This effect impairs Na^+^ and K^+^ active transport and plays a crucial role in the pathophysiological process from the early stages of the disease. The metabolic disorders from this process cause NEFA and bilirubin elevated serum levels and decreased albumin concentrations.

Albumin saturation and hypoalbuminemia likely increase the NEFA cytotoxic effects [80]. OA regulates fatty acid metabolism. Intravenous OA administration (40 mg per kg of body weight) lowers plasma NEFA concentrations, while higher doses are toxic and lead to lung injury. Conversely, results suggest a beneficial effect of lower doses of orally administered OA (about 40 to 80 mg per kg of body weight) in reducing plasma NEFA concentrations in normal animals [90].

FA, such as omega 3, can inhibit Na/K-ATPase activity in human endothelial cells [91]. They are also known for their effect on inflammation, with reports showing that high blood levels of NEFA overactivate the TLR/NF-κB pathway, promoting the expression of several inflammatory cytokines such as IL-1β, IL-6, and TNF-α [92]. Increased TNF production predicts poor clinical outcomes in patients with leptospirosis, with increased cytokine production associated with higher patient mortality levels during the disease progression. The increased production of inflammatory mediators in leptospirosis may be related to recognition mechanisms involving TLR4 and fatty acid receptors and a mechanism dependent on Na/K-ATPase signaling [84]. Results showed increased lung levels of these cytokines in BALF (bronchoalveolar lavage fluid) supernatants in GLP- or ouabain-challenged animals. This was the first report of lung injury induced by intra-tracheal ouabain injection [88].

Elevated levels of NEFA in the bloodstream, and an imbalance between NEFA and albumin levels, added to the fact that NEFA have pro-inflammatory capability due to the activation of TLR, may explain the pro-inflammatory status of leptospirosis. These observations might reveal key molecular targets to better understand leptospirosis pathophysiology and develop new treatments. Lung inflammation induced by GLP is not dependent on the activation of TLR4, reinforcing the role of Na/K-ATPase in this process. Even though inflammasome activation by low concentrations of intracellular K^+^ plays a critical role, GLP activates the p38 pathway, possibly through Na/K-ATPase, resulting in inflammation [57]. Na/K-ATPase can also be suggested as a primary OA target in the mechanism of ERK activation [93].

The relationship of oleic acid/albumin and the reestablishment to near control values was shown in preliminary studies to be an excellent parameter indicating leptospirosis patient recovery, demonstrating its potential as a prognostic biomarker factor for leptospirosis outcomes [94]. Due to its importance, a new test adapting three different methodologies to provide information concerning albumin–NEFA saturation determined by albumin isoelectric focusing and staining with bromocresol green was developed, providing an alternative to the current methodology for serum NEFA quantification with the advantage of measuring albumin’s ability to bind NEFA [95,96].

## 7. Na/K-ATPase Signalosome

More recently, knowledge of ionic homeostasis revealed the direct involvement of Na/K-ATPase in intracellular signaling pathways. Changes in cytoplasmic K^+^ concentrations are closely related to the transduction events implicated in apoptosis. Thus, different pathophysiological conditions with changes in the activity and/or cellular expression of Na/K-ATPase can trigger apoptosis signaling [97,98]. This phenomenon assumes greater complexity, as modification of enzymatic activity can occur either by changing the speed of regulation and/or cellular expression of its isoforms or by different effects on the functional modulation of the enzyme triggered by inhibitory and activating substances [99,100].

**Figure 2:**
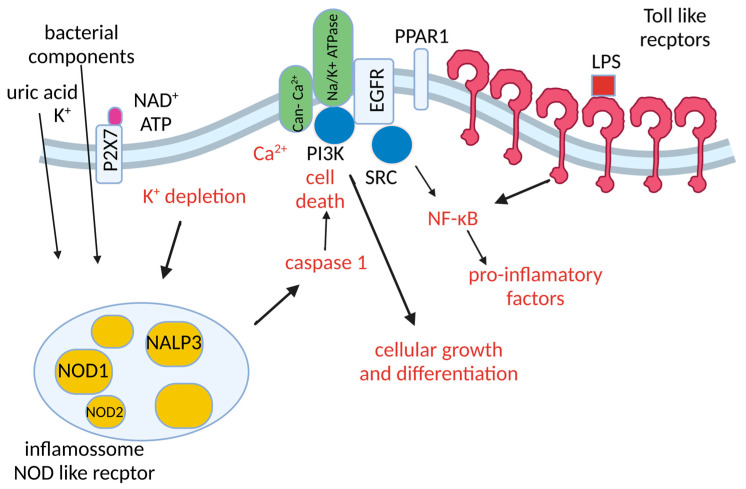
Na/K-ATPase signalosome. Na/K-ATPase can act as a signal transducer. GLP can activate multiple pathways. Activated Na/K-ATPase rapidly activates Src tyrosine kinase, which activates the EGFR or PI3K. Activated EGFR and PI3K can induce cell growth and differentiation, ROS production, and NF-κB activation. Na/K-ATPase signalosome activates calcium ion release into the cytoplasm. Calcium oscillation activates NF-κB. By inhibiting Na/K-ATPase activity, K^+^ decreases in the cytosol activation inflammasome NALP3, which can lead to cell death or increased inflammatory response. Src, non-receptor tyrosine kinases; EGFR, epithelial growth factor receptor; PI3K, phosphoinositide 3′ kinase; ROS, reactive oxygen species; NLR family pyrin domain containing 3 (NLRP3).

Therefore, in light of current knowledge, in addition to functioning as an ionic pump, Na/K-ATPase is a complex transducer pathway in cell signaling. It is assumed that, throughout evolution, the function of intracellular signaling is a pathway acquired through incorporating several interaction domains of the enzyme molecule with other proteins and/or ligands [101]. The enzyme can interact in caveolae with different signaling proteins, including Src kinase (which is a non-receptor tyrosine kinase), PKC, PKA, and PI3K [101,102], as well as caveolins [103]. Through the interaction of Na/K-ATPase with Src, EGFR (epidermal growth factor receptor), and other proteins, a transduction and signaling microdomain is formed, the signalosome (Figure 2) [68], restricted to the caveolae [104,105]. These observations strongly suggest that the activation of the complex formed by Na/K-ATPase–Src, from the interaction of ouabain with the enzyme, has a role in triggering EGFR and other intracellular signaling pathways.

Thus, ouabain binding to Na/K-ATPase regulates the interaction between the enzyme, caveolin, and cytoplasmic Src. In turn, activated Src activates EGFR, which recruits adapter proteins, following the activation of the Ras–Raf–ErK1/2 cascade [106,107]. These events promote changes in the expression of multiple genes and regulate intracellular Ca^2+^ concentration [106]. Intracellular Ca^2+^ oscillations, in turn, activate NF-kB, a pluripotent transcription factor, which activates genes that modulate cell proliferation, apoptosis, and the development of immune system responses. At the same time, Src activation also stimulates other pathways, including increased mitochondrial production of reactive oxygen species (ROS), which is an essential step in inducing apoptosis [108]. However, the pharmacological effect of cardiac glycosides on the intracellular signaling response is not homogeneous and depends on tissue type, exposure time, and drug concentration.

Interestingly, signal transduction promoted by Na/K-ATPase appears to occur through properties that are not dependent on its function as an ion pump [109,110], and the effect of ouabain on Src may occur independently of changes in intracellular Na^+^ and K^+^ concentrations [111]. In addition, Na/K-ATPase impacts cancer development by interfering with cell adhesion, motility, and migration in cancer cells [112] and cell apoptosis and autophagy [113]. It is also linked to virus replication [114,115].

### Signalosome in Leptospirosis

**Figure 3:**
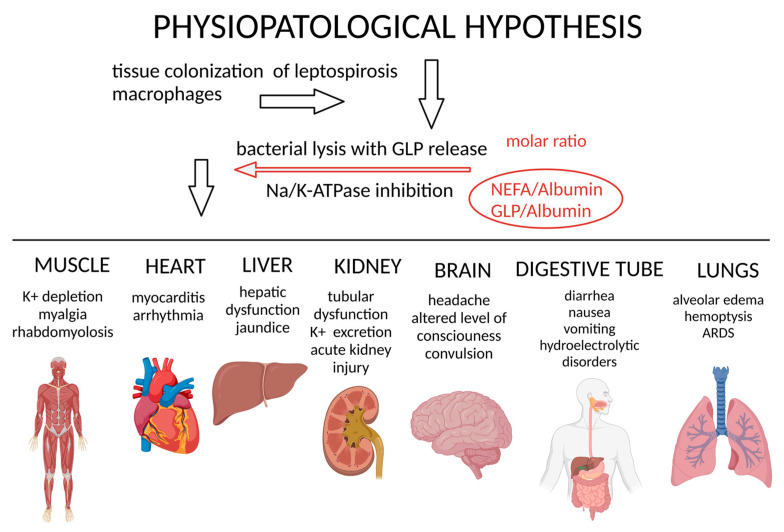
Overview of physiopathological hypothesis for leptospirosis. Infection with tissue colonization, macrophage leptospira lysis, and release of GLP with Na/K-ATPase inhibition can lead to multiple organ dysfunction.

More than thirty years ago, when the effect of Leptospira endotoxin (GLP) on the Na/K-ATPase enzyme was first described, the potent cellular transduction mechanisms linked to the functioning of the enzyme were not yet known. However, the exuberance of the clinical manifestations of leptospirosis already pointed to a pathophysiological mechanism distinct from the mechanisms known for other infectious diseases. The electrolyte disturbances and the peculiarity of the systemic inflammatory response of leptospirosis vary from mild clinical forms to the failure of multiple organs, present in Weil’s Disease. The relationship between the inhibition of the Na/K-ATPase enzyme and the cellular phenomena resulting from leptospirosis is illustrated in Figure 3.

## 8. Final Remarks

Based on the presented cellular events, the cellular pathophysiological involvement in leptospirosis was illustrated based on the events verified in the main organs affected by the disease. After penetration into the organism and following the leptospiremia phase, the bacterium has a preferential tropism for the kidneys and liver, where colonization and rapid multiplication occur by binary division. The greater the bacterial load in the tissues, the greater the source of GLP endotoxin that acts on the underlying cells. The infection activates the host’s defense system and generates bacterial lysis, exposing the endotoxin GLP, which integrates the cell wall of the Leptospira. The molar relationship between the bioavailability of GLP and the concentration of proteins present in the tissues, notably albumin, results in the local cytotoxicity potential of the GLP endotoxin.

The GLP lipototoxicity involves inhibiting Na/K-ATPase activity, a mechanism sufficient to cause many varied clinical manifestations in different organs and tissues. Enzymatic inhibition is clinically manifested according to the cells involved. In polarized epithelial cells, there is a dissipation of the transepithelial gradient, compromising the multiple functions of secretion and reabsorption dependent on the sodium gradient. In neurons and excitable cells, it compromises the generation and conduction of nerve impulses. In cells in general, GLP can activate intracellular pro-inflammatory signaling via the Na/K-ATPase signalosome. Activations of intracellular pathways promote the expression of pro-apoptotic genes, culminating in cell death. In addition to inflammatory status induced during leptospirosis, metabolic alterations occur with increased levels of NEFA-inducing lipotoxicity. Thus, GLP has the potential to induce inflammatory and metabolic alterations through Na/K-ATPase inhibition or triggering the signalosome, which might explain their cellular and clinical conditions. Future studies may help to unveil the role of Na/K-ATPase in Leptospirosis pathogenesis and as a potential therapeutic target for critically ill patients with Weil’s Syndrome.

## 9. Conclusions

Clinical manifestations of leptospirosis are due to the peculiarities of the cellular interactions promoted by Leptospira. The main feature is that Na/K-ATPase is the molecular target of the GLP endotoxin. GLP has a unique cytotoxic effect by inhibiting different isoforms of the Na/K-ATPase enzyme distributed in different cell types. Cellular disturbances caused in different organs colonized by the bacteria coincide with the clinical and laboratory manifestations present in the disease. Alongside this overshooting inflammatory state, metabolic alterations occur, and dyslipidemia stands out, with increased levels of circulating free fatty acids, causing an imbalance in the molar ratio of circulating fatty acids/albumin, triggering lipotoxicity events in various tissues. Synergistically, multiple molecular stimuli observed during infection activate the inflammasome and signalosome pathways. Inhibition of Na/K-ATP activity also appears to be the main trigger of pro-inflammatory and metabolic changes during leptospirosis disease.

## Data Availability

Not applicable.
